# Bilateral ischemic maculopathy in acquired immune deficiency syndrome

**DOI:** 10.1186/1869-5760-3-15

**Published:** 2013-01-15

**Authors:** Kiran Turaka, Rahul Reddy, Ashkahn Golshani, Wong Yu Khaw, J Shepard Bryan

**Affiliations:** 1 Associated Retina Consultants Ltd, 7600 N 15th Street, Suite #155, Phoenix, AZ, 85020, USA; 2 Banner Good Samaritan Medical Center, Phoenix, AZ, 85006, USA

**Keywords:** *Cryptococcus* meningitis, Human immunodeficiency virus, Immunosuppression, Ischemia, Maculopathy, Retinopathy

## Abstract

**Background:**

This brief report aims to report a case of bilateral macular ischemia as a cause of sudden decreased vision in a patient with acquired immune deficiency syndrome (AIDS).

**Findings:**

A 26-year-old male with disseminated cryptococcal meningitis, *Candida* thrush, *Pneumocystis jiroveci* pneumonia, and positive human immunodeficiency virus (HIV) infection with CD4 count of 4 cells/μl complained of sudden blurred vision in both eyes while on treatment with systemic antiviral, antifungal, and antibiotic medications. Ocular examination revealed HIV retinopathy changes with significant macular ischemia in both eyes, which was confirmed by fluorescein angiography. One dose of intravitreal foscarnet (1.2 mg/0.1 cc) was injected in both eyes. Laboratory work-up of serum and vitreous samples showed negative cytomegalovirus (CMV) titers. At 2 weeks of follow-up, he was started on treatment with atripla, a combination anti-retroviral therapy for AIDS. At 6 weeks of follow-up, there was an improvement in visual acuity and clinical findings.

**Conclusions:**

Noninfectious HIV retinopathy in AIDS is common, but bilateral macular ischemia is a rare presentation. It is important to rule out CMV retinitis as it is a major cause of visual morbidity among AIDS patients.

## Findings

### Introduction

Ocular manifestations in AIDS can be visually devastating due to associated infections [1]. In the early stage of human immunodeficiency virus (HIV) infection, noninfectious HIV retinopathy may be a cause of visual compromise [1]. Macular ischemia and edema are rare findings. Few authors reported clinical macular ischemia among AIDS patients [2-5]. We hereby report a case of symmetric bilateral macular ischemia in an AIDS patient. Patient's consent was obtained before all the tests were performed and for the reporting of this case in the medical literature.

### Case description

**Figure 1:**
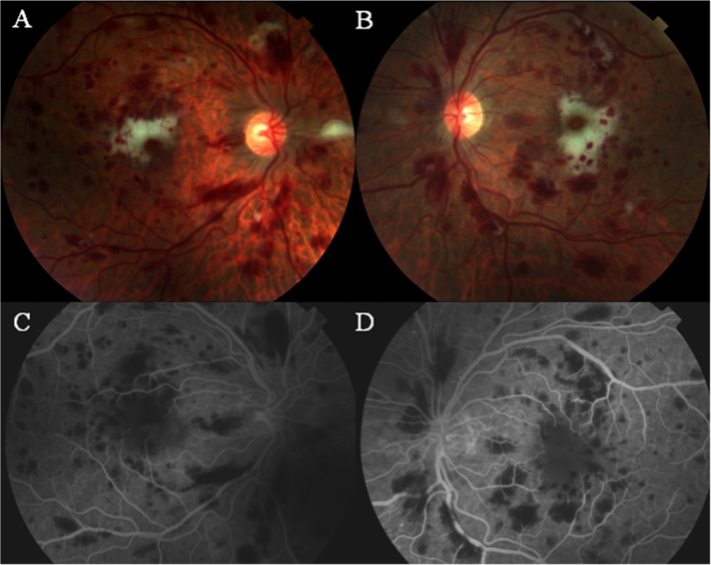
**Fundus examination at initial assessment.** Color fundus photograph of the right **(A)** and left eye **(B)** showing multiple intraretinal hemorrhages, cotton wool spots, and whitening in the perifoveal region. Fluorescein angiography (late phase) is showing the blocked areas corresponding to the hemorrhages in both eyes **(C, D)** and the enlarged foveal avascular zone with mild evidence of leakage around the perifoveal vessels.

**Figure 2:**
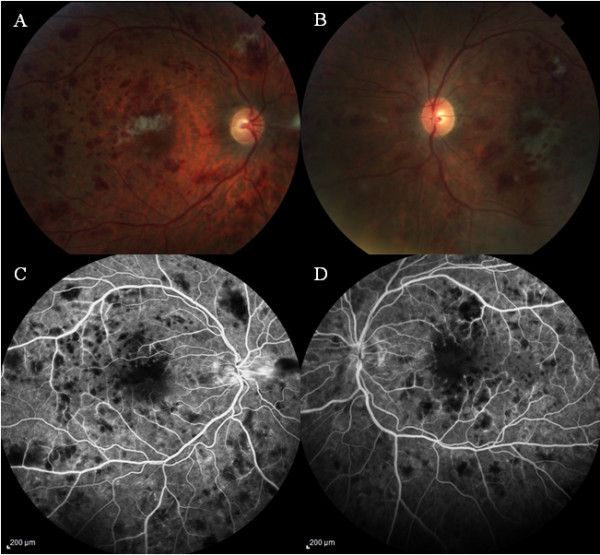
**Fundus examination at 6 weeks follow-up.** Color fundus photograph of the right **(A)** and left eye **(B)** showing mild decrease in the intraretinal hemorrhages, cotton wool spots, and perifoveal whitening. Fluorescein angiography is showing the blocked areas corresponding to the hemorrhages **(C, D)** in late phase with mild decrease in the enlarged foveal avascular zone in both eyes compared with Figure 1C,D.

A 26-year-old Caucasian man was referred to rule out cytomegalovirus (CMV) retinitis after a complaint of blurred vision. He had medical history of *Pneumocystis jiroveci* pneumonia (PJP), *Candida* oral thrush, and cryptococcal meningitis 1 month prior to presentation and was on continuing treatment with intravenous ganciclovir (5 mg/kg), fluconazole (800 mg), azithromycin (1,200 mg), and atovaquone (1,500 mg). His laboratory work-up revealed positive serology for HIV-I, PJP, and cryptococcal antigen and negative serology for herpes simplex virus (HSV) I/II, CMV, RPR, hepatitis A/B/C, cocci IgG/IgM, AFB, *Chlamydia* and gonococcus, and *Toxoplasma* IgG. CD4 count was 4 cells/μl. He had acute anemia and thrombocytopenia which was treated by 11 units of packed red blood cells and four units of platelets. Visual acuity was counting fingers (CF) 2 ft in both eyes (OU). Intraocular pressure (IOP) was 10 mmHg in the right eye (OD) and 8 mmHg in the left eye (OS). Slit lamp biomicroscopic (SLB) examination revealed unremarkable anterior segment and no vitritis in OU. Fundus examination of OU showed optic disc hyperemia, multiple superficial and intraretinal hemorrhages, and cotton wool spots in the posterior pole. There was marked subfoveal whitening in the macula (Figure 1A,B) and areas of clinical ischemia in the peripheral retina of OU. Fluorescein angiography (FA) revealed areas of blocked fluorescence corresponding to the intraretinal hemorrhages in early phases. There was significant symmetrical blocked fluorescence in the fovea of both eyes with increase in foveal avascular zone and mild leakage along perifoveal capillaries (Figure 1C,D) in late phase. Diagnosis of ischemic maculopathy secondary to HIV-induced occlusive vasculitis was made. Vitreous and aqueous samples were obtained for serologic testing. He was treated with intravitreal foscarnet 1.2 mg/0.1 ml in both eyes for the suspected CMV retinitis. At 1 week of follow-up, the visual acuity improved mildly to 4/200 in the OD and CF in the OS. IOP was 6 mmHg in OU. There was no change in the anterior segment or fundus findings in OU compared with the initial examination. Laboratory results of the vitreous/aqueous samples were negative for PCR, CMV, HSV, cocci, and toxoplasmosis titers. Optical coherence tomography showed a central foveal thickness of 361 μ in the OD and 345 μ in the OS. At this point, we did not administer additional intravitreal foscarnet injections and followed him with close observation. The infectious disease specialist delayed highly active anti-retroviral therapy (HAART) due to the recent history of cryptococcal meningitis which might lead to immune reconstitution inflammatory syndrome. He was continuing systemic ganciclovir, fluconazole, azithromycin, and atovaquone. At 2 weeks of follow-up, systemic ganciclovir was discontinued, and the patient was started on HAART (atripla) therapy. At 6 weeks of follow-up, visual acuity had improved to 20/400 in the OD and 20/200 in the OS, and IOP was 12 and 10 mmHg in the OD and OS, respectively. SLB examination of the anterior segment was unremarkable in OU. Fundus examination in OU showed mild optic disc pallor and mild decrease of the intraretinal hemorrhages and cotton wool spots in the posterior pole, with mild decrease in subfoveal whitening in the macula (Figure 2A,B) of OU. FA revealed areas of blocked fluorescence corresponding to the intraretinal hemorrhages in the posterior pole in early phase and also a symmetrical blocked fluorescence in the fovea of both eyes with decrease of the increased foveal avascular zone (Figure 2C,D) in late phases.

## Discussion

HIV retinopathy is the most common ocular manifestation in AIDS, but CMV retinopathy is the major cause of visual morbidity [1,2]. Noninfectious HIV retinopathy leading to visual disability was reported among AIDS patients. Superficial and intraretinal hemorrhages, cotton wool spots, and small/large vessel occlusions are the common clinical findings in HIV retinopathy; macular ischemia and/or edema were considered rare findings [1-6]. The three hypothesis in the pathogenesis of HIV retinopathy as described by Jabs were immune complex deposition, HIV infection of the retinal vascular endothelium, and hemorrhagic abnormalities [1]. Our patient initially presented with multiple systemic disseminating infections and, after extensive work-up, was diagnosed with HIV infection with low CD4 count of 4 cells/μl. While recovering from cryptococcal meningitis, his decreased visual acuity raised suspicion of CMV retinitis. Clinical ocular examination showed signs of HIV retinopathy with evidence of symmetrical perifoveal ischemia, which was confirmed by FA. There were no signs of uveitis, vitritis, or major retinal vasculitis in either eye of our patient. Cunningham and associates described bilateral macular disease among four out of five patients who presented with vision loss [2]. Ninety percent of them presented with either active or inactive CMV retinitis, whereas our patient showed no evidence of prior active retinitis and had negative serology for CMV. They felt that localized vascular occlusions secondary to CMV retinitis might be the cause for vision loss [2]. All of their patients were receiving HAART, whereas our patient was not on HAART at initial presentation. Another interesting etiology for macular ischemia described by Yoganathan and associates in a 41-year-old HIV-positive man was zidovudine (AZT)-induced anemia that led to bilateral hemorrhages and macular ischemia which was thought be due to anemia of AZT toxicity after ruling out other etiologies [5]. There was improvement in ocular symptoms and signs after discontinuing AZT at 1 year follow-up [5]. Bui and coauthors reported that drug-induced pancreatitis could be a cause of abnormal retinal findings such as Purtschner retinopathy among the AIDS patients [7]. Our patient had no signs and symptoms of pancreatitis and feels that multiple factors like immunosupression due to systemic infections, immune-mediated HIV retinopathy, and possibly severe anemia might have caused the abnormal fundus findings.

Treatment of HIV retinopathy with intravitreal ganciclovir (injections and implants) and foscarnet was found to be effective [8,9]. Our patient initially was treated with intravitreal foscarnet injection, but with negative CMV titers of vitreous/aqueous samples, we did not administer further local therapy.

In conclusion, our patient had bilateral macular ischemia at presentation. Without much ocular intervention while on systemic treatment, he regained reasonable vision. Though we had initial suspicion for associated CMV retinitis, negative serology made us decide not to give additional intravitreal treatment. Continued systemic treatment had improved his systemic and ocular disease status.

## Competing interests

The authors declare that they have no competing interests.

## Authors’ contributions

KT and RR were involved in treating the patient, wrote the manuscript, and made corrections after receiving comments from the coauthors. AG and WYK were involved in the medical care of the patient, also reviewed the paper, and gave valuable comments from their perspective. JSB was involved in the vigorous review of the paper and made the best possible comments. All authors read and approved the final manuscript.
